# Covid-19 vaccination intensions among literate Ghanaians: Still the need to dissipate fear and build trust on vaccine efficacy?

**DOI:** 10.1371/journal.pone.0270742

**Published:** 2022-06-29

**Authors:** Joseph Agebase Awuni, Michael Ayamga, Gilbert Dagunga

**Affiliations:** 1 Department of Economics, University for Development Studies, Tamale, Ghana; 2 Department of Applied Economics, University for Development Studies, Tamale, Ghana; 3 Department of Science Education, St. John Bosco College of Education, Navrongo, Ghana; Groupe ESC Dijon Bourgogne, FRANCE

## Abstract

**Purpose:**

The study examined Covid-19 vaccinations intentions among literate Ghanaians and how it is been influenced by vaccine mistrust and the fear of the unforeseen side effects.

**Design/Methodology/Approach:**

We used cross sectional data collected from 223 respondents by means of questionnaire disseminated through social media from 16^th^ to 20^th^ April, 2021. Likert-scale questions were asked regarding the knowledge, attitudes and perceptions of literate Ghanaians towards COVID-19 vaccines. Kruskal-Wallis and sample t-test were performed to ascertain the differences in vaccination intentions between key socioeconomic variables. A pairwise correlation was performed to examine the relationship between vaccination intensions and fear of the unforeseen, mistrust of the vaccine and concerns of profiteering. Finally, a binary probit regression model was fitted to examine the predictive effect of key variables on respondent’s vaccination intentions.

**Findings:**

The results revealed a relatively low level of knowledge about the safety and efficacy of the COVID-19 vaccines. The sample t-test showed that males have a relatively positive attitude towards the COVID-19 vaccines than females at 5% level of significance. Mistrust of vaccine safety and efficacy have a significant negative influence on vaccination intensions at 1% significance level.

**Originality/Value:**

This study provides the Ghanaian government and other stakeholders with useful information to aid in educational campaigns on the safety and effectiveness of the COVID-19 vaccine. More campaign efforts towards females could help increase uptake given their relatively poor attitudes towards the vaccine.

## 1. Introduction

The coronavirus disease (COVID-19) pandemic has adversely impacted years of progress in global development and has further compounded the challenges of inequality, poverty and food insecurity especially for developing countries. According to the World bank [1], about 100million more people have been pushed into extreme poverty due to the pandemic, the highest rate experienced in over 20years. The pandemic has revealed several structural weaknesses in developing countries evident in higher rates of inflation, fuel prices, and exchange rate depreciation among others [1]. Globally, as of 4^th^ March, 2022, there have been 440,807,756 309 confirmed cases of COVID-19, including 5,978,096 deaths [2]. In Ghana, the first case of COVID-19 was reported on 12^th^ March, 2020. As of 4th March, 2022, there were 160,028 confirmed cases of COVID-19 with 1,442 deaths in Ghana [2]. The pandemic compelled countries all over the world to adopt a sequence of emergency measures and management mechanisms [3]. The Government of Ghana imposed a number of social distancing measures and other protocols to help curtail the spread of the infection including an unprecedented three weeks partial lockdown of the two biggest cities (Accra and Kumasi). An estimated 42,000 people lost their jobs in the first two months of the pandemic in Ghana with the tourism sector alone losing $171 million dollars due to the partial lockdown and closure of tourism and hospitality centers [4]. It is generally agreed that, the long-term success of public health measures against the COVID-19 pandemic will depend on acquired immunity in a sufficient proportion of the population (herd immunity), which necessitate mass vaccination [5]. By 8^th^ March, 2021, Ghana’s Food and Drugs Authority (FDA), had granted Emergency Use Authorization (EUA) to two COVID-19 vaccines for use in Ghana; Oxford-AstraZenica/Covishield (Serum Institute of India) and Sputnik V Vaccines (Generium Joint Stock Company, Russia) [6]. On 24^th^ of February 2021, Ghana became the first country to receive about 600,000 doses of the Oxford-AstraZeneca COVID-19 vaccines through the WHO led pooled global procumbent mechanism known as the COVAX scheme [7]. The country received a second batch of 350,000 doses of the Oxford/AstraZeneca COVID-19 vaccines on the 7^th^ of May 2021. Subsequently, the Johnson and Johnson and Moderna vaccine brands were also procured. The government of Ghana aimed at vaccinating at least 60% of the estimated 31 million residents by close of 2021 in order to attain herd immunity. Despite the availability of vaccines doses, as of 29^th^ March 2022, a total of 5, 070, 36 individuals were fully vaccinated, representing about 16.3% of the total targeted population and still far less than the expected 60% expected to have been vaccinated by close of 2021 [8]. This suggests somewhat a high resistance or hesitancy of the population to vaccinate. [9] found that about 40.7% of Ghanaians were not willing to take the covid-19 vaccine from a survey of 108 responses. Similar study by [10] showed that, about 45.9% of Ghanaians were not willing to take the vaccine in 2020. [11] revealed that the low level of vaccination was attributable to individual hesitancy to take the vaccine and that contributed to some 12,780 doses of covid-19 vaccines getting expired in the Volta region of the country. In February 2022, the Ministry of Health attempted to implement a mandatory vaccination policy for all Ghanaians which was widely resisted [12]. The question then is, why are people resisting or hesitating to take the vaccine? Could it be reasons ascribed to fear of vaccine safety and mistrust?

The WHO has stated that vaccine hesitancy is one of the top 10 significant threats to global health [7]. Wide acceptability of vaccines by the general public is therefore very crucial for populations to attain herd immunity against the Covid-19 pandemic [13].

According to [14], literacy is the ability to identify, understand, interpret, create, communicate and compute, using printed and written materials associated with varying contexts. In developing countries, literate members of society often serve as reliable sources of information on issues such as COVID-19 to the illiterate segment of the population [15]. Therefore, their vaccination intensions are vital for policy since the illiterate ones are expected to follow the example of the literate. Also, one’s intension for a behaviour is influenced by their knowledge, attitudes and perceptions (KAPS) of the action. This is particularly important for developing countries like Ghana where majority of the population hesitates in taking the vaccine. For instance, a systematic review of covid19 vaccine hesitancy worldwide by [13] showed low level of acceptance in Africa. It is against this background that the present study sought to examine the vaccination intentions of literate Ghanaians towards the COVID-19 vaccines as well as estimate the predictive effect of individual perception about vaccine safety (trust), perceived unforeseen side effects and perceptions of profiteering on vaccination intentions. The findings of this study could therefore help the Ghanaian authorities to fine tune campaign messages for wide acceptance of the covid-19 vaccines.

## 2. Materials and methods

### 2.1 Research setting

Ghana is the second largest economy in West Africa after Nigeria. In 2020, the GDP of Ghana was 74.26 billion US dollars [16]. There are about 15 Public universities in Ghana, 10 Technical universities, 44 colleges of Education and other private tertiary institutions. Adult literacy rate in Ghana (the percentage of adults aged 15 years and above who can both read and write in English, the national language) was 79% in 2018 [17]. In terms of health care facilities, there were 5,421 Community-based Health Planning and Services (CHPS) Compounds, 998 clinics, 140 district hospitals, 1,004 health centres, and 357 hospitals as of 2017. Health facilities and health workers in Ghana are disproportionately found in the Ashanti and Greater Accra regions, especially in urban areas [18]. In terms of critical health staff (such as community health nurses, enrolled nurses, medical officers, and pharmacist, among others), Ghana has 90,703 personnel, out of which 13,438 (14.8%) and 13,120 (14.5%), respectively, are found in Ashanti and Greater Accra regions (Ghana Health Service, 2018)

### 2.2 Study design and participants

The study used a cross sectional data collected from 223 respondents by means of snow ball sampling techniques. The self–administered questionnaire was designed using google forms and disseminated through social media (WhatsApp platforms) of health workers, university lecturers, students, and teachers among others. This approach was most appropriate because of COVID-19 restriction and protocols that were imposed by the government to minimize the spread. To be included in the study, the individual must be resident in Ghana and should be able to read and respond to the questionnaire without requiring any assistance. Participation in the study was voluntary and anonymity was ensured by the design of the questionnaire. The period of data collection was from 16^th^ to 20^th^ April, 2021. The total responses received during this period was then used for the study.

### 2.3 Instruments and measurement

The questionnaire for the study was developed based on the instruments of recent empirical studies on the subject of study including those of [19] and [5]. The draft questionnaire was pre-tested with three students and two health workers to examine their comprehension of the questions. All the questions were retained after the pre-test. The final version of the questionnaires contained 23 questions; seven (7) questions on the respondent demographic and socioeconomic characteristics (Age of respondent, gender, location of hometown, primary occupation, field of specialty, level of study and usage of the internet), Five (5) questions on COVID-19 related knowledge questions, six (6) questions on attitude related questions and five (5) questions on perception.

#### 2.3.1 COVID-19 related knowledge

The section sought to determine the knowledge of respondents regarding the COVID-19 vaccine. Hence 5 questions related to knowledge of the existence of the vaccine in the country, the effectiveness of the vaccine, vaccination without knowledge of COVID-19 status, allergic reactions and knowledge whether vaccine boost immune system against other infectious diseases were asked. Participants were asked to indicate a “Yes”, “No” or “Don’t Know” to the statements been asked.

#### 2.3.2 Attitude towards COVID-19 vaccines

A three-point likert scale (1 = Disagree, 2 = Indifferent and 3 = Agree) was used for participants to indicate the level at which they disagree or agree to 6 attitudinal questions about the vaccine. Hence statement related to vaccine safety, confidence of protection, intensions to vaccinate, need to encourage others to vaccinate among others as presented in the appendix.

#### 2.3.3 Perceptions about COVID-19 vaccines

Another set of 4 questions on participants perception about the vaccine were asked. Participants were asked to indicate either “Yes”, “No” or “Maybe” to these statements pertaining to the perception about the side effects of the vaccine, perception about others profiteering from vaccines for financial gain other than for people’s health.

### 2.4 Statistical analysis

The data was analyzed using Microsoft Excel 2019 and STATA 15 package. For knowledge, the maximum possible score is expected to be 5, for attitude the maximum is expected to be 6 and a maximum score of 4 for the perception variable. Following [19], the responses were recoded such that every positive response to knowledge related item was coded 1 and if otherwise, 0. Also for attitude, a positive attitude was coded 1 and if otherwise, 0 while for perceptions, a negative attitude was coded 1, otherwise, 0.

Categorical variables were analyzed using frequencies and percentages while continuous variables were analyzed by means and standard deviation. Kruskal-Wallis test was used to test for the difference in knowledge, attitudes and perceptions between key socioeconomic variables. [20] indicated that, in determining whether there are statistically significant differences between two or more groups of an independent variable, the Kruskal Wallis test is more suitable because it does not assume a normal distribution of the underlying data. Also, the sample t-test was used to determine the group difference with knowledge, attitude and perception scores.

The Pearson’s correlation coefficient was used to analyze the correlation between the knowledge, attitude and perceptions of participants. Finally, a binary probit model was fitted to ascertain the effect of COVID-19 knowledge and perceptions (i.e., Trust, unforeseen effects and perception of profiteering) on their vaccination intensions. Vaccination intentions (Appendix C, question 5) was recategorized into a binary outcome, where an individual is assigned a code of 1 if they agree that they will vaccinate without hesitations, otherwise 0.

Since the dependent variable is dichotomous, we first assume y* to be an underlying continuous latent variable that makes an individual willing to vaccinate, the latent variable could be modeled as;

$$y^{*}=X\beta +\varepsilon$$

Where X is a vector of variables that could influence vaccine hesitancy including fear of the unforeseen, mistrust and profiteering concerns.

Hence *Y_i_* = 1 if y*>0 and *Y_i_* = 0 if y*<0

The probit model is then given as;

$P_{i}=\Phi (y^{*})=\Phi (X\beta +\varepsilon )=P_{i}=F(X\beta +\varepsilon )$

Where F is the standard normal cumulative distribution function which can be written as

$$F(X\beta )=\frac{1}{\sqrt{2\pi }}\int_{-\infty }^{X\beta }e^{-Z\frac{2}{2}}dz$$

The average marginal effect after the probit model was then fitted in order to interpret probabilities at which these variables affect vaccination intentions.

### 2.5 Ethical considerations

All procedures for the study were carried out under the ethical guidelines of the University for Development Studies (UDS) research ethics and approved by the covid-19 committee before the study began. Informed consent was solicited from Participants in writing before the administration of the questionnaires.

## 3. Results

### 3.1 General characteristics of participants

A total of 223 respondents were included in the final analysis. Table 1 presents the general characteristics of respondents in the survey. The results show that most (68.61%) of the participants were males with 31.39% being females. In terms of the occupation, students were in the majority of the respondents constituting about 29.15% followed by teachers at 25.56%, then health workers (17.48%), University lecturers (15.25%) and others (12.56%). About 48.37% of the respondents had formal education up to an undergraduate level. Those educated up to the masters and PhD levels were 29.77% and 6.05% respectively. Some 15.81% of the respondents had formal education up to the diploma level. A majority (84.30%) of the respondents were regular internet users, while about 15.7% were occasional users of the internet (Table 1).

**Table 1:** General characteristics of respondents.

Variable	Frequency	Percentage (%)
***Gender***		
Male	70	68.61
Female	153	31.39
***Primary Occupation***		
Student	65	29.15
Teacher	57	25.56
Health worker	39	17.48
Lecturers	34	15.25
Others	28	12.56
***Field of Educational Specialization***		
Social Science	89	39.91
Agricultural Science	50	22.42
Health Science	46	20.63
Others	38	17.04
***Level of study***		
Undergraduate	104	48.37
Masters	64	29.77
Diploma	34	15.81
PhD	13	6.05
***Use of Internet***		
Most of the time	188	84.3
Sometimes	35	15.69

### 3.2 Knowledge about the COVID-19 vaccine

Table 2 presents the distribution of knowledge items among participants by gender. As shown in Table 2, the Kruskal Wallis test produced a statistically significant difference between males and females in terms of knowledge about the COVID-19 vaccines in the country with 96.08% of females and 100% of males indicating that they knew about the COVID-19 vaccines. A relatively higher percentage of males (48.68%) knew about the effectiveness of the COVID-19 vaccines as against 37.68% of females (Table 2). On the whole, about 45.2% of the respondents reported knowing about the effectiveness of the vaccine, which is an indication that majority (54.8%) of the respondents did not know about the effectiveness of the COVID-19 vaccines. About 47.98% of respondents believed it was dangerous to vaccinate without knowing one’s COVID-19 status, while 23.98% thought that taking the vaccines could increase allergic reactions.

**Table 2:** Distribution of each knowledge item by gender difference.

Variable	Total		Male		Female		chi2	df	p-value
	n	%	n	%	n	%			
Do you know about COVID-19 Vaccine?									
Yes	216	97.3	147	96.08	69	100	2.768	2	0.0963*
No	4	1.80	4	2.61	0	0			
Don’t Know	2	0.9	2	1.31	0	0			
Do you know about the effectiveness of COVID-19 Vaccine?									
Yes	100	45.2	74	48.68	26	37.68	0.3260	2	0.5682
No	88	39.82	51	33.55	37	53.62			
Don’t Know	33	14.93	27	17.76	6	8.70			
It is dangerous to get vaccinated without knowing your COVID-19 status									
Yes	107	47.98	68	44.44	39	55.71	0.3890	2	0.5328
No	71	31.84	58	37.91	13	18.57			
Don’t Know	45	20.18	27	17.65	18	25.71			
Does vaccination increase allergic reactions?									
Yes	53	23.98	38	25.17	15	21.43	0.519	2	0.4713
No	46	20.81	32	21.19	14	20			
Don’t Know	122	55.2	81	53.64	41	58.57			
Does vaccination boost immune system against other infectious diseases?									
Yes	63	28.38	44	28.95	19	27.14	0.713	2	0.3983
No	63	28.38	46	30.26	17	24.29			
Don’t Know	96	43.24	62	40.79	34	48.57			

* Represents 10% significance level.

### 3.3 Attitude towards COVID-19 vaccine

Table 3 presents results on the attitude of respondents towards COVID-19 vaccines in Ghana. The results show a statistically significant difference between males and females on four items of the attitude scale including, attitude towards vaccine safety, confidence of vaccine protection, vaccination intentions as well as willingness to encourage family/friends to take vaccines.

**Table 3:** Distribution of each attitude item by gender difference.

Variable	Total		Male		Female		chi2	df	p-value
	n	%	n	%	n	%			
The COVID-19 vaccines are safe									
Disagree	49	21.97	33	21.57	16	22.86	6.400	2	0.0114**
Indifferent	107	47.98	66	43.14	41	58.57			
Agree	67	30.04	54	35.29	13	18.57			
I am confident of being protected after vaccination									
Disagree	63	28.38	32	21.05	31	44.29	2.794	2	0.0946*
Indifferent	84	37.84	58	38.16	26	37.14			
Agree	75	33.78	62	40.79	13	18.57			
I will take the COVID-19 vaccine without any hesitation if it is made available to me									
Disagree	65	29.15	40	26.14	25	35.71	3.955	2	0.0467**
Indifferent	63	28.25	40	26.14	23	32.86			
Agree	95	42.6	73	47.71	22	31.43			
I will also encourage my family/friends/relatives to get vaccinated									
Disagree	60	26.91	40	26.14	20	28.57	2.830	2	0.0925*
Indifferent	69	30.94	43	28.10	26	37.14			
Agree	94	42.15	70	45.75	24	34.29			
It is not possible to reduce the incidence of COVID-19 without being vaccinated									
Disagree	91	40.8	61	39.60	30	42.86	0.468	2	0.4941
Indifferent	54	24.22	36	23.53	18	25.71			
Agree	78	34.98	56	36.60	22	31.43			
It is not possible to get infected with COVID-19 after being vaccinated									
Disagree	117	52.7	76	50	41	58.57	0.199	2	0.6556
Indifferent	63	28.38	44	28.95	19	27.14			
Agree	42	18.92	32	21.05	10	14.29			

*and *** represents 10% and 5% significance level respectively.

About 30.04% of all the respondents agreed that the vaccines were safe. Segregating by gender shows 35.29% of males and 18.57% of females agreed that the vaccines were safe. More males (40.79%) were confident of vaccine protection than females (18.57%). Meanwhile, 47.71% and 31.43% of males and females respectively indicated willingness to take the vaccine without hesitation when it is made available to them. That means that, 52.28% of males and 68.57% of females were either indifferent or not willing to vaccinate. Similarly, 45.75% of the males and 34.29% of females indicated willingness to encourage family and friends to take the COVID-19 vaccine. About 34.98% of the respondents indicated that it was possible to reduce the incidence of COVID-19 without being vaccinated and only 18.92% indicated that it was possible for one to get infected after being vaccinated.

### 3.4 Perception of participants towards COVID-19 vaccine

The perception of participants towards COVID-19 vaccines is presented in Table 4. It shows that there was statistically significant difference between males and females on only one item ‘*others promoting the vaccine for financial gain than people’s health’*.

**Table 4:** Distribution of each perception item by gender difference.

Variable	Total		Male		Female		chi2	df	p-value
	n	%	n	%	n	%			
Do you think the COVID-19 vaccine may have side effects?									
Yes	158	70.85	108	70.59	50	71.43	0.002	2	0.9656
No	6	2.69	6	3.92	0	0			
Maybe	59	26.46	39	25.49	20	28.57			
Do you think that if everyone in the society will keep to the prevention measures, COVID-19 pandemic can be eradicated without vaccination?									
Yes	124	55.61	81	52.94	43	61.43	1.070	2	0.3010
No	36	16.14	27	16.76	9	12.86			
Maybe	63	28.25	45	28.49	18	25.71			
Do you think some people in Ghana are profiteering from the COVID-19 vaccines?									
Yes	134	60.36	92	60.53	42	60	0.000	2	1.000
No	20	9.01	13	8.55	7	10			
Maybe	68	30.63	47	30.92	21	30			
Do you think some authorities are promoting vaccinations for financial gain rather than people health?									
Yes	116	52.25	71	46.41	45	65.22	6.469	2	0.0110**
No	29	13.06	22	14.38	7	10.14			
Maybe	77	34.68	60	39.22	17	24.64			

On the whole, about 70.85% of the respondents perceive that the COVID-19 vaccine may have side effects. While 55.61% perceive that COVID-19 pandemic could be eradicated without vaccinations if everyone would adhere to the preventive measures. About 60.36% believe that some people in the country are profiteering from the COVID-19 vaccines, while 52.25% thinks that some people are promoting the COVID-19 vaccines for financial gain rather than health reasons.

### 3.5 Group difference analysis (bivariate) with knowledge, attitude and perception scores

We further performed a sample t-test to examine the differences in knowledge, attitude and perception between key variables (i.e., gender, educational level and working status) under the study. The scores for respondents were generated for all three areas of knowledge, attitude and perceptions as presented in Table 5.

**Table 5:** Group difference analysis (bivariate) with knowledge, attitude and perception scores.

Variable	Knowledge about Vaccine M(SD)	t-value	Attitude towards vaccine M(SD)	t-value	Perception about vaccine M(SD)	t-value
*Average Score*	1.14(1.17)		2.64(1.84)		1.28(1.06)	
*Gender*		-0.6702				
Male	1.30(1.17)		2.84 (1.87)	2.446**	1.33(1.05)	1.149
Female	1.41 (1.17)		2.20(1.71)		1.15(1.08)	
*Educational level*						
Diploma	0.91 (1.16)	1.9858**	2.812 (1.77)	-1.226	1.14 (1.02)	-2.194**
Undergraduate	1.406 (1.18		3.29 (1.99)		1.65(1.20)	
Masters	1.23(1.09)	0.6412	2.18 (1.68)	-3.775***	1.26 (1.04)	-0.630
PhD	1.45 (1.18)		4.07 (1.89)		1.46 (1.05)	
*Primary Occupation*						
Workers	2.11(1.75)	-2.274**	3.21(2.49)	-2.713***	1.29 (1.03)	0.665
Students	1.18 (1.15)		2.17(1.65)		1.15(0.84)	

M = mean, SD = Standard deviation while ** and *** represents 5% and 1% significance level respectively.

The average knowledge score was found to be 1.14 with a standard deviation of 1.17 suggesting that, out of the five knowledge items considered, a respondent on the average is likely to correctly respond in 1 or 2 of items suggesting some appreciable knowledge about the covid-19 vaccine. The average score for attitude was about 2.64(SD = 1.84) suggesting a low to moderate positive attitude among the respondents. Meanwhile, an average perception score of 1.28(SD = 1.06) was recorded suggesting that, on the average respondents had negative perception in 1 or 2 of the 4 perception items asked.

The results show that, there was statistically significant difference in attitude towards the COVID-19 vaccines between males and females with the males recording a relatively higher (M = 2.84) positive attitude than the females (M = 2.20). Such results further confirm the earlier results in Table 3. The respondents who had PhD qualifications and those who had a job at the time of the survey had a better positive attitude towards the COVID-19 vaccine than those with lower qualifications and students respectively.

### 3.6 Relationship between vaccination intentions, fear of the unforeseen, mistrust and concerns on profiteering

A Pairwise correlation was performed to ascertain if there was a relationship between vaccination intensions, fear of the unforeseen, mistrust of the vaccine and concerns of profiteering. The results in Table 6 show that respondents vaccination intensions (Measured as 1 if respondents indicated he/she was willing to take the vaccine without hesitation and 0 if otherwise) was negatively associated with respondent fear of the unforeseen side effects, mistrust of the vaccine and concerns of profiteering. There was a strong negative association between respondents’ willingness to vaccinate and mistrust.

**Table 6:** Pairwise correlation between vaccination intentions, fear of the unforeseen, mistrust and concerns on profiteering.

Variable	Vaccination Intentions	Fear of unforeseen	Mistrust	Concerns on Profiteering
Vaccination Intentions	1	-	-	-
Fear of the unforeseen	-0.0261	1	-	-
Mistrust	-0.6222	0.0114	1	-
Concerns on Profiteering	-0.1127	0.1221	0.0451	1

We further conducted a binary probit regression to examine the predictive effect of these variables and some socio-demographic variables on respondent’s vaccination intentions in Ghana. The definition of these variables and how they were measured is showed in Table 8 at the appendix. The results of the probit estimates are presented in Table 7. Mistrust of COVID-19 vaccine was found to have a negative influence on respondents’ willingness to take a vaccine. Respondents who do not trust the COVID-19 vaccines are 67.5% less likely to vaccinate against COVID-19 relative to those who trust the COVID-19 vaccines. Also, health workers were more willing to vaccinate as compared to other non-health workers.

**Table 7:** Predictive effect of perceptions about fear of the unforeseen, mistrust of vaccine and concerns of profiteering on vaccination intentions.

Vaccination Intentions	Coef.	St.Err.	Marginal Effects (dy/dx)	Std.Err.
Age	.013	.034	0.003	0.008
Gender	-.528	.41	-0.127	0.096
Location	.136	.487	0.033	0.118
Health_Occ	1.185**	.582	0.286**	0.128
Lecturer	.646	.609	0.160	0.149
Student	.434	.521	0.107	0.129
Higher_Education	.386	.474	0.095	0.117
Internet_often	-.336	.489	-0.084	0.122
Unforseen_future	-.253	.388	-0.063	0.096
mistrust	-3.4***	.464	-0.675**	0.053
Profiteering	-.501	.37	-0.123	0.091
Constant	2.009	1.31		
N = 223 Pseudo R2 = 0.3397 LR chi2 = 103.36 Prob(chi2) = 0.000				

** and *** represents 5% and 1% significance level respectively.

## 4. Discussion

The cross-sectional study examined vaccinations intentions of literate Ghanaians. We examined the knowledge, attitudes and perceptions of literate Ghanaians towards the covid-19 vaccines. The results showed a relatively low level of knowledge about the safety and effectiveness of the COVID-19 vaccine among the respondents. Even though about 97.30% of the respondents were aware of the presence of the vaccine in the country, they were very doubtful about the effectiveness of the COVID-19 vaccine, and felt that it was dangerous for someone to receive the vaccine without knowing his/her COVID-19 status. Over 50% of the respondents were either indifferent or did not agree that vaccines were safe or offered protection against Covid-19. This suggest a need for more sensitization and educational campaigns about the safety and efficacy of the COVID-19 vaccine if the government’s aim of vaccinating at least 60% of the population is to be achieved. In neighboring Nigeria, [19] reported that vaccine skepticism could undermine efforts aimed at ending the COVID-19 pandemic. Similar results have been reported in other developing countries outside sub-Saharan Africa. For instance, [21] also reported a low level of knowledge regarding the effectiveness of COVID-19 vaccines in Bangladesh.

Male respondents had a relatively higher positive attitude towards the COVID-19 vaccines than females. There was widespread misinformation on various social media platforms that taking Covid-19 vaccines could lead to women not being able to conceive or become pregnant in the future. Similar results were found in previous studies [22]. Such results suggest a need for the Ghanaian authorities to consider campaign messaging that could dispel such misinformation and encourage more female participation in the ongoing vaccination exercise if the country is to achieve the vaccination target of at least 60% of the population. This is particularly important since females form the majority of the Ghanaian population. The results revealed that on the whole, higher educational attainment was associated with a more positive attitude towards the COVID-19 vaccines.

The study found a considerably high level of negative perception about the COVID-19 vaccine among the respondents. For example, about 70.85% of the respondents perceive that the vaccines could have some hidden or unknown long-term side effects. About 60.36% of the respondents had the perception that, some people in the country were profiteering from the COVID-19 pandemic, in particular, 52.25% of the respondents indicated that some powerful individuals were promoting the vaccine for their selfish financial gains. An individual perception influences his or her actions and the relatively high negative perception about the vaccine in the country needs to be corrected in order to achieve the target of vaccinating at least 60% of the entire population.

About 42.60% of the respondents indicated willingness to receive the COVID-19 vaccines without any hesitation when it is made available to them. The remaining 57.40% were either not willing or indifferent on their vaccination intentions. A pairwise correlation analysis revealed that, participant’s willingness to take the COVID-19 vaccine was negatively correlated with the fear of the unforeseen (side effects). There was a strong negative correlation between vaccination intensions and mistrust of vaccines.

A binary probit model further confirmed that, mistrust of vaccines had a negative and significant effect on respondents’ vaccinations intentions at 5% level of significance. Such results suggest that respondents who did not have confidence in the safety of the COVID-19 vaccines were unwilling to take the vaccine. The probability that those who mistrusted the vaccine efficacy and less likely to take the vaccine was 67.50% more than those who trusted it ceteris paribus. Our results are consistent with that of [5] who found that mistrust of COVID-19 vaccine affected vaccination intentions in the UK. There is therefore the need to boost trust and confidence among Ghanaian residents on the safety and efficacy of the COVID-19 vaccine to instill trust and improve the willingness to vaccinate.

## 5. Conclusion and recommendation

The study examined the literate Ghanaians vaccination intentions against COVID-19 as well as estimated the effect of mistrust of vaccines, the fear of the unforeseen future side effects and concerns about profiteering and individual vaccination intentions. Using a data collected from 223 respondents, we employing a mixed-method in the analysis. Results from the study revealed a relatively low level of knowledge about the safety and efficacy of the COVID-19 vaccines. There are some significant negative perception and attitude about the COVID-19 vaccines. Male respondents showed a relatively more positive attitude and were more likely to receive the COVID-19 vaccines than female respondents. Mistrust about the vaccine safety was found to adversely affect respondent’s vaccination intentions. There is therefore the need for the Ghanaian and civil society groups to intensify campaigns and educational messages about the safety and effectiveness of the COVID-19 vaccines approved for use in the country. This will enlighten citizenry and erase some of the wrong notions people hold about the vaccine in the country. More vaccination campaign efforts targeting females is particularly necessary to help increase uptake given the relatively poor attitudes to the vaccines compared with the males.

## 6. Limitations of the study

First, the data was collected through social media platforms and there is no guarantee that participants did not search for answers from other sources which can introduce some bias. To minimize this bias, the period in which the data was taken was limited to 5 days. Also, the method in which the data was taken limited the participation of illiterates. Further research on the subject should consider increasing the sample to include both literates and illiterates.

## Appendix

### UNIVERSITY FOR DEVELOPMENT STUDIES

We are a team of researchers from the University for Development Studies, collecting data for purely academic purpose. The research seeks to examine the vaccination intensions among the literate Ghanaian. Questions related to the Knowledge, Attitudes and Perceptions (KAP) towards covid-19 vaccines are asked. ***Your responses would be anonymous and confidential***

**A. DEMOGRAPHIC CHARACTERISTICS**

1. Age of respondent……………
2. Sex of respondent Male [] Female []
3. Which part of Ghana do you come from? North [] South []
4. Which of the following categories does your programme of study falls under?
  Health Science [] Natural & Agric Science [] Social Science [] Others []
5. What is the level of your study? Diploma [] Undergraduate [] Masters [] PhD []
6. How often do you use the internet? Not at all [] Sometimes [] Most often []

**B. COVID-19 Vaccine Related Knowledge**

**Please indicate Yes (Y) or No (N) or Don’t know (D) to the following statements/questions**

1. Do you know about the COVID-19 vaccine?
2. Do you know about the effectiveness of the COVID-19 vaccine?
3. It is dangerous to get vaccinated without knowing your COVID-19 status
4. Does vaccination increase allergic reactions?
5. Does vaccination boost immune system against other infectious diseases?

**C. ATTITUDES TOWARDS COVID-19 VACCINES**

**Indicate the level in which you agree to the following. In each case 1 = Disagree, 2 = Indifferent/undecided 3 = Agree**

1. The COVID-19 vaccines are safe
2. I am confident of being protected after vaccination
3. I will take the COVID-19 vaccine without any hesitation if it is made available to me
4. I will also encourage my family/friends/relatives to get vaccinated
5. It is not possible to reduce the incidence of COVID-19 without being vaccinated
6. It is not possible to get infected with COVID-19 after being vaccinated

**D. PERCEPTIONS TOWARDS COVID-19 VACCINES**

1. Do you think the COVID-19 vaccine may have side effects? Yes [] No []
2. Do you think that if everyone in the society will keep to the prevention measures, COVID-19 pandemic can be eradicated without vaccination? Yes [] No []
3. Who do you think should first be vaccinated? (Select only 3)
  Politicians []
  Health workers []
  Teachers and students []
  The Aged and people with underlying health conditions []
  Commercial drivers []
  General public []
4. Do you think some people in Ghana are profiteering from the COVID-19 vaccines?
  Yes [] No []
5. Do you think some authorities are promoting vaccinations for financial gain rather than people health? Yes [] No []

**Table 8:** Definition of variables and measurement.

Variables	Measurement
Age	Years
Gender	Dummy (1 if male, otherwise 0)
Location	Dummy (1 if northern Ghana, otherwise 0)
Health_Occ	Dummy (1 if health worker, otherwise 0)
Lecturer	Dummy (1 if yes, otherwise 0)
Student	Dummy (1 if student, otherwise 0)
Higher_Education	Dummy (1 if undergraduate and above, otherwise 0)
Internet_often	Dummy (1 if most often, otherwise 0)
Unforseen_future	Dummy (1 if respondent fear unforeseen future, otherwise 0)
mistrust	Dummy (1 if respondent mistrust vaccine, otherwise zero)
Profiteering	Dummy (1 if respondent perceive authorities are profiteering from the vaccines, otherwise 0)

## Supporting information

S1 File (DTA)
